# In situ architecture of the nuclear pore complex of the higher plant *Arabidopsis thaliana*

**DOI:** 10.1038/s41477-025-02138-y

**Published:** 2025-10-31

**Authors:** Ingrid Berenice Sanchez Carrillo, Patrick C. Hoffmann, Agnieszka Obarska-Kosinska, Victor Fourcassié, Martin Beck, Hugo Germain

**Affiliations:** 1 Department of Biochemistry, Chemistry, Physics and Forensic Science, Université du Québec à Trois-Rivières, Trois-Rivières, Quebec Canada; 2 Department of Molecular Sociology, Max Planck Institute of Biophysics, Frankfurt am Main, Germany; 3 Proteomics Platform, Centre de recherche du CHU de Québec, Faculty of Medicine, Université Laval, Québec City, Quebec Canada; 4 Institute of Biochemistry, Goethe University Frankfurt, Frankfurt am Main, Germany

**Keywords:** Nuclear receptors, Plant cell biology

## Abstract

The nucleus is enclosed by the nuclear envelope, which contains nuclear pore complexes (NPCs). While NPCs have been well studied in vertebrates, yeast and algae, in situ structural data for higher plants is lacking. Here we show that individual nucleoporins of *Arabidopsis thaliana* and humans exhibit high structural similarity. We report an in situ NPC structure of higher plants, derived from *A.* *thaliana* root protoplasts using cryo-electron tomography, subtomogram averaging and homology-based integrative modelling. We present the AtNPC model based on predictions of *A.* *thaliana* nucleoporins (NUPs), supported by mass spectrometry. Here the AtNPC scaffold contains one Y-complex ring at the cytosolic and two at the nuclear ring. The AtNPC contains prominent NUP155 connector elements that are conserved in human NPCs but not in *Chlamydomonas reinhardtii* NPCs. Our model suggests that the ELYS homologue HOS1 plays an important role in the head-to-tail connection of Y-complexes in AtNPCs.

## Abstract (web summary)

Using advanced imaging techniques and computer modelling, the authors were able to create a detailed structural model of a nuclear pore complex from a seed plant.

## Main

Nuclear pore complexes (NPCs) are large multiprotein complexes involved in the selective import and export of macromolecules passing through the nuclear envelope^1^. Exclusive to eukaryotic organisms, NPCs play crucial roles in regulating gene expression^2^, chromatin organization^3^, DNA repair, RNA processing and quality control^4,5^. NPCs are organized into different subcomplexes^6^ made of multiple copies (~1,000 protein subunits in humans^7^) of approximately 30 different proteins known as nucleoporins (NUPs)^8^. With a few exceptions, NUPs are largely conserved among eukaryotes^7^.

Much of our understanding of the NPCs comes from various studies using electron microscopy (EM)^9^, which has been continuously further developed and refined, allowing the study of a broad range of model species^10–16^. Cryo-electron tomography (cryo-ET) studies have revealed that the NPC forms a three-layered scaffold structure^7,14,15,17,18^, with an octagonal symmetry around the central transport channel^19^. These three layers consist of the three main rings: the nuclear ring (NR), situated on the nucleoplasmic side, the inner ring (IR), embedded at the fusion point of the inner and outer nuclear membrane^7^, and the cytoplasmic ring (CR) facing the cytoplasm. Structural data for the NPCs of organisms such as yeast^10,20,21^, *Chlamydomonas reinhardtii*^13^, *Xenopus laevis*^15,22,23^ and human^24^ are now available, showcasing a range of structural differences within their NPC scaffolds. However, technical hurdles have limited the use of cryo-ET for higher plants and our understanding of plant NPCs is largely based on mass spectrometry experiments^12^ and NUP homology with well-characterized organisms. Recently, we optimized *Arabidopsis thaliana* root protoplast sample preparation for cryo-ET^25^, which we use here to gain insight into the structure of the higher plant NPC.

*A.* *thaliana* is a small rosette plant that was first adopted as a model organism for its utility in genetic studies. It features a quick generation time, a small size (minimizing the need for extensive growing facilities), ease of genetic transformation and prolific seed production through self-pollination. All of this makes it a great model for plant biology^26,27^. In contrast to many organisms, *A.* *thaliana* can withstand a high level of homozygosity and has a relatively small genome (132 Mbp)^28,29^. Despite its short life cycle, producing transgenic plants still requires several months^30^. To circumvent these delays, electroporation and polyethylene glycol-based transfection of protoplasts have grown in popularity as tools for the transient expression of genetic material. Protoplasts have been extensively used to study various aspects of plant physiology, cell ultrastructure and genetics^31^. The procedure of enzymatically removing the cell wall does not obscure cell type differences or prevent comparisons with whole tissues as it preserves physiological responses and cellular activities^32–34^. While procedures have been developed for employing protoplasts for various purposes, the use of in situ cryo-ET to study plant protoplasts was still limited until recently.

In this study, we prepared vitrified *A.* *thaliana* root protoplasts by cryo-focused ion beam (cryo-FIB) milling for cryo-ET^35^, and combined it with subtomogram averaging (STA) to examine and reveal the NPC structure from a higher plant within its cellular environment. We constructed a model for the *A.* *thaliana* NPC (AtNPC) based on the structure prediction of *A.* *thaliana* NUP homologues using integrative modelling. We identify a unique NPC arrangement and conformation for the AtNPC, which we then compare with the unicellular algae *C. reinhardtii* (CrNPC) cryo-ET map^13^ and the *Homo sapiens* NPC structural model (HsNPC)^14^.

## Results

### Mass spectrometry reveals most NUPs of the NPC in roots

To identify the NUPs that make up the AtNPC we performed nuclear extraction^36^ from root protoplasts followed by high-performance liquid chromatography and tandem mass spectrometry. Our results allowed us to identify the majority (31) of the currently reported *A. thaliana* NUPs^37–39^ in the root protoplast nuclei preparation (Table 1). Among the NUPs identified in our study, we detected all NUPs of the inner ring and the NUPs of the Y-complex. NUP50a, NUP136, CG1, NUP98b, GBPL3 and CPR5 could not be detected, with a false discovery rate (FDR) of 1%. NUP50a, NUP136 and GBPL3 are nuclear basket proteins^37,40^, while CG1 is a peripheral NUP that interacts with the cytoplasmic side of the NPC^41^. NUP98b is an FG-NUP located to the peripheral sides of the central channel^3^. A plausible explanation for the undetected peripheral NUPs is that some may have dissociated from the NPC during the nuclear extraction and sample preparation. CPR5 however is a transmembrane NUP located in the nuclear envelope^42^ and should remain at the nuclear envelope during preparation. However, both CPR5 and NUP98b were also not detected in *A.* *thaliana* seedlings by proximity-labelling mass spectrometry in another recent study^43^. Overall, our workflow demonstrated that we were able to detect most NUPs of the nuclear basket, the core NUPs and cytosolic NUPs, allowing us to further use this information to gain a better understanding of which NUPs form the NPC in *A.* *thaliana* protoplasts.

**Table 1:** Mass spectrometry identification of AtNUPs from nuclear extracts purified from *A.* *thaliana* root protoplasts

Protein	Accession number	emPAI value Sample 1	emPAI value Sample 2	emPAI value Sample 3	s.d. Sample 1	s.d. Sample 2	s.d. Sample 3	Presence
ALADIN	AAAS_ARATH	0.4000	0.3560	0.1987	0.1267	0.1746	0.0782	3/3
NUP50C	Q93ZH3_ARATH	0.0914	0.0000	0.0000	0.0033	0.0000	0.0000	1/3
HOS1	HOS1_ARATH	0.5453	0.7713	0.6147	0.0792	0.0665	0.0990	3/3
NUP205	A0A1P8BGZ1_ARATH	0.5220	0.5757	0.6167	0.1031	0.1561	0.1902	3/3
GP210	GP210_ARATH	0.9063	1.3200	1.0507	0.3410	0.0300	0.1028	3/3
NUP35	NUP35_ARATH	0.1230	0.1827	0.0000	0.0046	0.0809	0.0000	2/3
NUP43	NUP43_ARATH	0.1127	0.4867	0.2793	0.1115	0.5187	0.0588	3/3
NUP50B	NU50B_ARATH	0.0603	0.0000	0.0000	0.1045	0.0000	0.0000	1/3
NUP54	NUP54_ARATH	0.1773	0.5193	0.0000	0.0601	0.1638	0.0000	2/3
NUP58	NUP58_ARATH	0.1572	0.0867	0.0182	0.0782	0.0891	0.0315	3/3
NUP62	NUP62_ARATH	0.3720	0.6040	0.0821	0.1005	0.1459	0.0037	3/3
NUP82	F4K465_ARATH	0.0171	0.0589	0.0361	0.0296	0.0585	0.0016	3/3
NUP85	NUP85_ARATH	0.6697	1.0183	0.6583	0.1230	0.2886	0.2657	3/3
NUP88	NUP88_ARATH	0.1689	0.1087	0.1398	0.0645	0.0586	0.0632	3/3
NUP93A	NP93A_ARATH	0.7823	0.6067	0.3477	0.0926	0.3361	0.1736	3/3
NUP93B	NU93B_ARATH	0.1094	0.0654	0.0948	0.0777	0.0277	0.0839	3/3
NUP96	NUP96_ARATH	0.1643	0.3200	0.1400	0.0609	0.1416	0.0320	3/3
NUP98A	NU98A_ARATH	0.0542	0.0764	0.0189	0.0235	0.0568	0.0164	3/3
NUP107	NU107_ARATH	0.1710	0.2847	0.2340	0.0879	0.0081	0.0187	3/3
NUP133	NU133_ARATH	0.2017	0.3237	0.2163	0.0736	0.1061	0.0097	3/3
NUP155	NU155_ARATH	0.6537	0.7207	0.4023	0.0672	0.1554	0.0668	3/3
NUP160	NU160_ARATH	0.2690	0.2323	0.3240	0.0501	0.1061	0.0901	3/3
NUP214	NP214_ARATH	0.0000	0.0080	0.0155	0.0000	0.0139	0.0160	2/3
NUA	NUA_ARATH	0.4960	0.6093	0.4250	0.1025	0.1793	0.0267	3/3
NUP188	F4JUG3_ARATH	0.1155	0.1032	0.0177	0.0360	0.0560	0.0069	3/3
NDC1	Q8LAF4_ARATH	0.2993	0.1712	0.1066	0.0589	0.0878	0.0930	3/3
GLE1	GLE1_ARATH	0.0000	0.0000	0.0135	0.0000	0.0000	0.0233	1/3
RAE1	RAE1_ARATH	0.4657	1.0457	0.1330	0.0692	0.9646	0.0454	3/3
SEH1	SEH1_ARATH	0.4093	0.6237	0.2433	0.1678	0.2010	0.0663	3/3
SEC13A	SC13A_ARATH	0.5030	0.1993	0.1254	0.1828	0.0907	0.0595	3/3
SEC13B	SC13B_ARATH	1.7567	0.9040	0.6103	0.5021	0.2272	0.1463	3/3

List of AtNUPs identified using mass spectrometry. The last column indicates in how many of the three technical replicates the protein was detected in. Accession numbers were obtained from the Uniprot database (https://www.uniprot.org/). emPAI values were determined by carrying out a Mascot search (Matrix Science) and analysis with the Scaffold software (version 5.2.2, Proteomes Software Inc.).

### Structural models of individual NUPs of the Y-complex are conserved

**Fig. 1:**
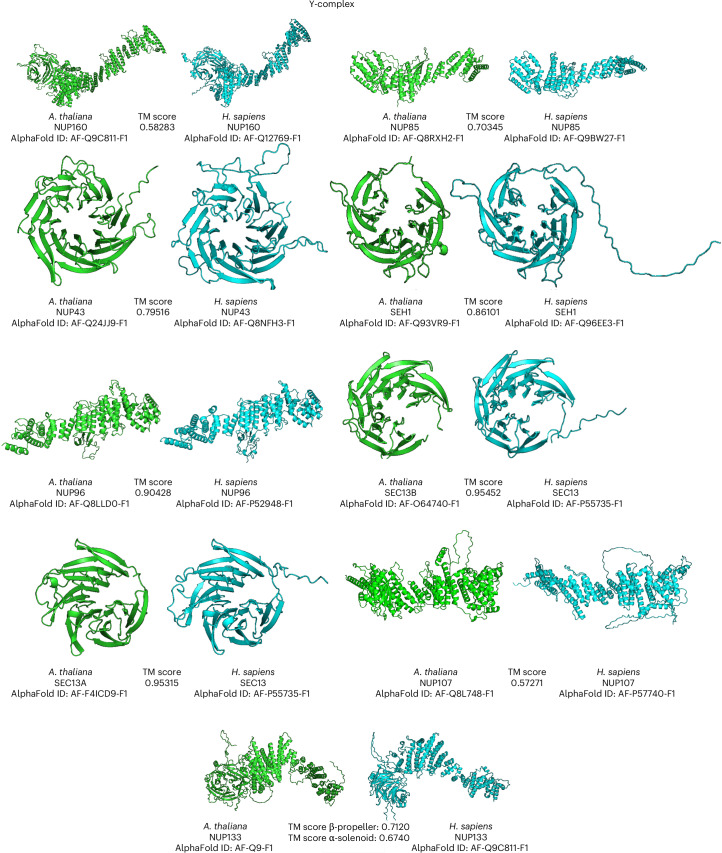
Comparison of AlphaFold-predicted structures of the NUPs that constitute the Y-complex in *H.* *sapiens* and *A.* *thaliana.* Side-by-side visualization of the AlphaFold-predicted tertiary structures of Y-complex NUPs in both *A.* *thaliana* (green) and *H.* *sapiens* (blue). The characterized elements are represented as helices and beta sheets and the uncharacterized regions are represented as lines. All files were downloaded from the AlphaFold Protein Structure Database (https://alphafold.ebi.ac.uk/), accessed and visualized with PyMOL. TM scores were calculated for NUPs present in the Y-complex by comparing *A.* *thaliana* with *H.* *sapiens* using TM-align^56^. ID accessions were obtained from the AlphaFold database (https://alphafold.ebi.ac.uk/ (refs. ^54,55,99^)). Owing to the flexible connection between the β-propeller and α-solenoid of NUP133, we calculated the TM scores for each domain separately.

Of the constituents that make up the NPC, the Y-complex (also known as the NUP84 complex in yeast or the NUP107–NUP160 complex in vertebrates) is a prominent and structurally well-characterized NPC substructure^9,44^. Consisting of six to ten NUPs depending on the organism^45^, this complex is a main constituent of the CR and NR^7^. Previous studies across different organisms^13,22,44,46–52^ revealed the conservation of proteins and the architecture among eukaryotic species^4,5,53^. We compared the three-dimensional (3D) structures of NUPs comprising the *A.* *thaliana* Y-complex, which were also present in our mass spectrometry data, with those predicted of the human Y-complex to visualize structural similarities between individual NUPs. To achieve this, we compared the AlphaFold predictions^54,55^ of the *A.* *thaliana* and *H.* *sapiens* Y-complex NUPs side by side (Fig. 1) and measured the Template Modelling (TM) score^56^ to assess their structural similarity. Such 3D structure comparisons present a means of gaining a better understanding of their evolution and function^57^.

Our results confirmed that some components of the Y-complex that are found in vertebrates are also broadly conserved in plants, as previously suggested^58^. We found that the *A.* *thaliana* Y-complex shares nine out of ten NUPs (NUP160, NUP85, NUP43, SEH1, NUP96, SEC13, NUP107, NUP133 and HOS1/ELYS) with that of *H.* *sapiens*. Based on the sequence analysis, the functional homologue of ELYS in plants is HOS1 (ref. ^37^), which we also detected by mass spectrometry. HOS1 has been reported to act as an E3 ubiquitin ligase and to regulate flowering in *A.* *thaliana*^37,59^. The remaining NUP, NUP37 in humans, is absent from plant NPCs in both *A.* *thaliana* and in apple. These findings suggest that, despite more than 500 million years of evolutionary separation between plant and animal cells, most of the Y-complex is conserved.

### In situ NPC structure of plant cells revealed in *A. thaliana*

To study plant NPCs in their native state, we utilized our recently established workflow^25^ employing isolated root protoplasts from transgenic *A.* *thaliana* plants expressing RAE1–GFP^37^ for in situ cryo-ET. In brief, we vitrified the isolated protoplasts on cryo-EM grids and the intact nuclei protoplasts expressing RAE1–GFP were then identified using cryo-correlative light and electron microscopy (cryo-CLEM). Protoplast lamellae were prepared by cryo-FIB milling. We specifically targeted areas of the nuclear envelope for cryo-ET tilt-series acquisition, followed by tomogram reconstruction and STA. These methods allowed us to structurally analyse the *A.* *thaliana* NPC in situ.

**Fig. 2:**
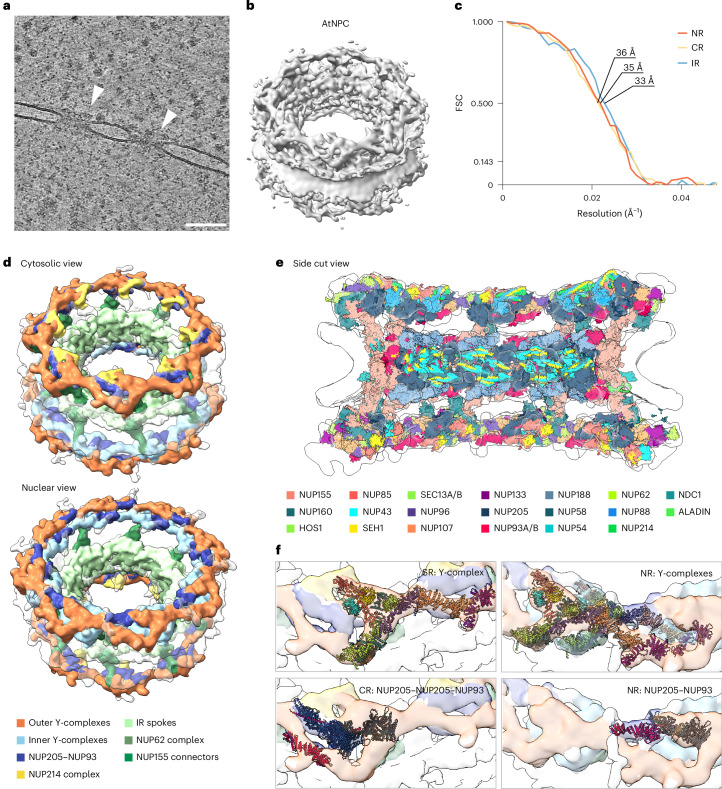
Subtomogram average and structural model of the AtNPC from root protoplasts. **a**, A tomogram slice showing the nuclear envelope with two embedded AtNPCs (white arrowheads). Scale bar, 100 nm. The image is extracted from the volume of a reconstructed tilt series; 111 tilt series were acquired. **b**, STA composite map of the AtNPC resolved to ~35 Å for the individual rings. **c**, Corresponding Fourier shell correlation (FSC) curves for the individual asymmetric subunits of the IR, NR and CR (FSC cutoff 0.143). **d**, Segmentation of the composite AtNPC from the cytosolic (top) and from the nuclear (bottom) view highlighting one Y-complex ring (orange) at the CR and two Y-complex rings at the NR (orange and light blue), the NUP214 complex (yellow) and NUP205–NUP93 (dark blue). The IR spokes are shown in light green with the central channel facing NUP62 complexes shown in green. Connectors between both CR and NR to the IR are shown in dark green. **e**, A structural model of the AtNPC built from predicted models of 20 NUPs, of which their presence was confirmed by mass spectrometry. **f**, Zoomed-in views on one spoke of the CR and NR displaying the structural arrangement of Y-complexes and of NUP205/NUP93. The colour code for NUPs is shown in the figure.

We acquired 37 tomograms of *A. thaliana* nuclear envelopes, which contained a total of 79 NPCs (Fig. 2a). Using a previously established STA approach^16,53^, we obtained the cryo-ET map of the *A.* *thaliana* NPC from these 79 NPCs at a resolution of 35 Å for the focused maps of individual rings (Fig. 2b,c). To better understand the eightfold symmetrical scaffold architecture, we segmented the cryo-ET map into its subcomplexes. On the cytosolic side, the CR of the *A.* *thaliana* NPC consists of one ring with eight copies of the Y-complex. In addition, each asymmetric unit contains density for the NUP214 complex and two NUP205–NUP93 complexes (Fig. 2d,f). In contrast, the NR contains two copies of the Y-complex per asymmetric unit and density for one NUP205–NUP93 complex, resulting in a total of 16 Y-complexes for the NR that form two concentric rings of inner and outer Y-complexes (Fig. 2d,f). Both CR and NR show prominent densities for NUP155 connectors linking them to the eight spokes of the IR (Fig. 2d). Having established the overall arrangement of the *A.* *thaliana* NPC, we constructed a structural model of a higher plant NPC. This model is based on our cryo-ET map and uses predicted models^54^ of 20 *A.* *thaliana* NUPs (Fig. 2e), which we had already confirmed by mass spectrometry to be present in root protoplasts (Table 1). While the structural model covers large areas of map density, unassigned map density remains in the regions where we expect the nuclear basket and the cytoplasmic filaments to bind to the NPC scaffold.

### *A. thaliana* connector elements resemble those of the HsNPC

**Fig. 3:**
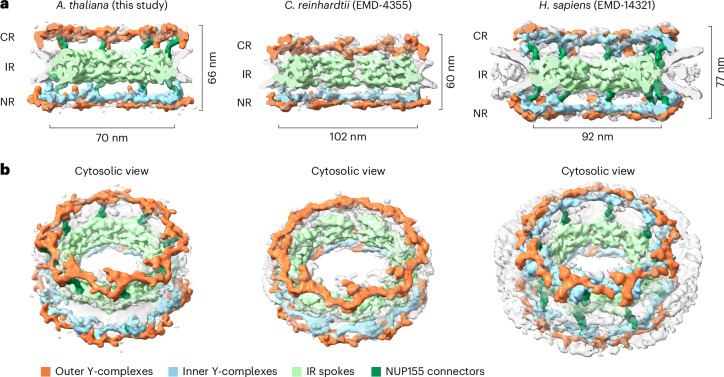
In situ NPC cryo-ET map of the scaffold architecture from *A.* *thaliana* root protoplasts in comparison to the cryo-ET maps of CrNPC and HsNPC. **a**, The side cut view of AtNPC, CrNPC and HsNPC and the diameter measured at the point of inner and outer nuclear membrane fusion, as well as the height of the NPC scaffold. **b**, Tilted views on the cytosolic side of the AtNPC, CrNPC and HsNPC. All views are shown at the same scale.

The individual subcomplexes comprising the CR, IR and NR of the NPCs are mostly conserved across different species^60^. Similarly, the AtNPC subcomplexes exhibit high structural similarity to previously published NPC building blocks^13^. To better illustrate the similarities of the AtNPC scaffold and to highlight potential differences to the NPC structures of other species, we compared our AtNPC cryo-ET map with the in situ NPC cryo-ET map of the single-cell algae *C.* *reinhardtii* (CrNPC) (EMD-4355 (ref. ^13^)) and the human in situ NPC cryo-ET map from HEK293 cells (HsNPC) (EMD-14321 (ref. ^14^)) (Fig. 3). Beyond the apparent similarities in the overall architecture, some differences are evident between the AtNPC, the CrNPC and the HsNPC.

**Extended Data Fig. 1:**
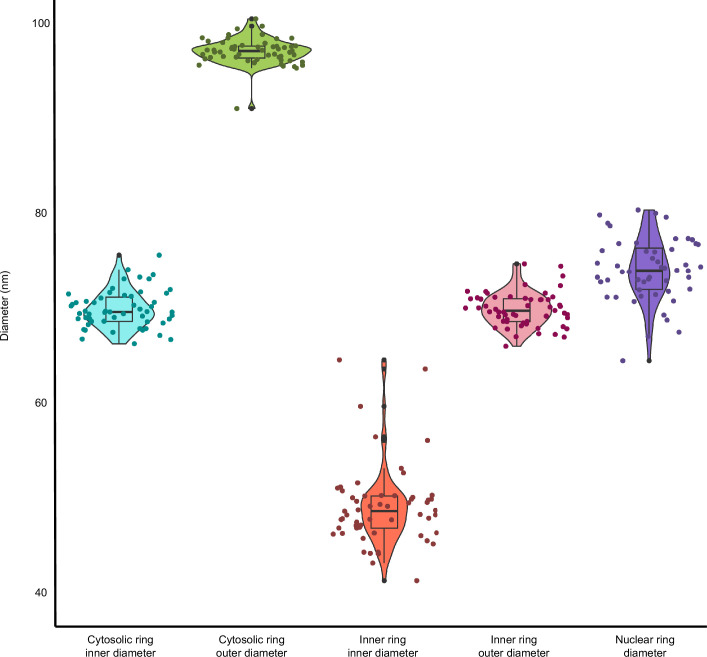
Variations in measurements of NPC diameter components. The violin plot presents the individual measurements taken for *A. thaliana* NPC and plotted for each ring. The box plot presents minimum and maximum values, median and interquartile range.

In comparison with the HsNPC, which features a symmetrical Y-complex arrangement with two concentric rings for both the CR and NR, the AtNPC Y-complexes are asymmetric with one ring at the CR and two rings at the NR. The presence of the connector elements (NUP155) between the outer rings and the IR, which are not clearly resolved in the CrNPC^13^, are prominent in the AtNPC (Fig. 2e). In terms of dimensions, AtNPCs are taller in height than the CrNPC, but shorter compared with HsNPCs. We also observe variations in the average diameter of the NPC (measured from membrane to membrane) across the three organisms. The AtNPC has the smallest average diameter, measuring approximately 70 nm between the nuclear envelope membrane. HsNPCs from HEK293 cells measure an average diameter of 92 nm and CrNPCs have the largest average diameter of 102 nm (Fig. 3). In all NPCs of the dataset, we observe variations in measured NPC diameters in a similar range as observed for other organisms (Extended Data Fig. 1). These differences in average diameter between the three organisms probably reflect the combination of inherent structural properties of the NPC and other external influences on the structure, such as membrane tension, osmolarity and nucleocytoplasmic transport^16,61,62^. However, to what extent these variations in diameter measurements and potentially their dynamics reflect functional or evolutional structural properties remains challenging to disentangle.

**Fig. 4:**
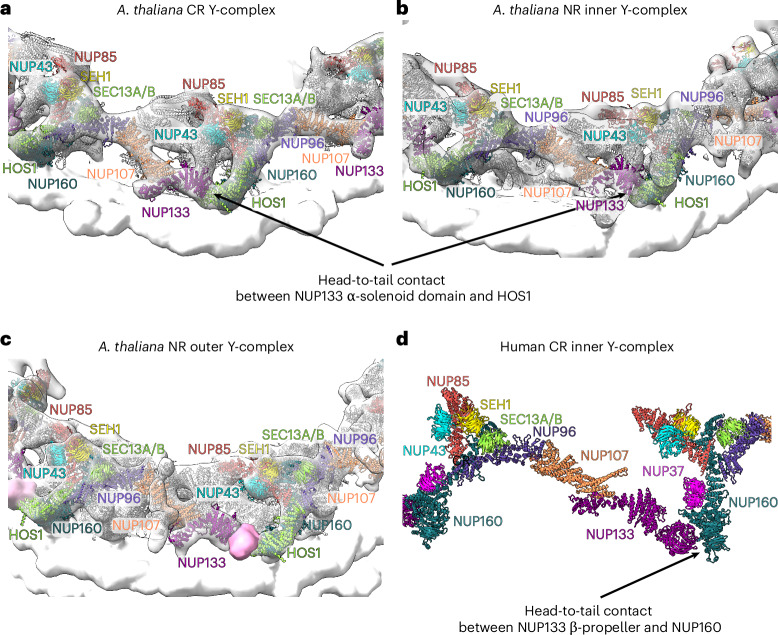
Head-to-tail contact of the Y-complexes. **a**, The CR of the AtNPC. **b**, The NR inner Y-complex of the AtNPC. The interface between NUP133 α-solenoid and HOS1 in **a** and **b** was predicted by AlphaFold2. **c**, The NR outer Y-complex of the AtNPC. The density shown in pink may correspond to the NUP133 β-propeller, but it was not explicitly modelled since AlphaFold2 did not predict this interaction. **d**, The inner Y-complex of the CR of the human NPC (PDB 7R5J)^14^. NUPs are coloured as in Fig. 2.

**Extended Data Fig. 2:**
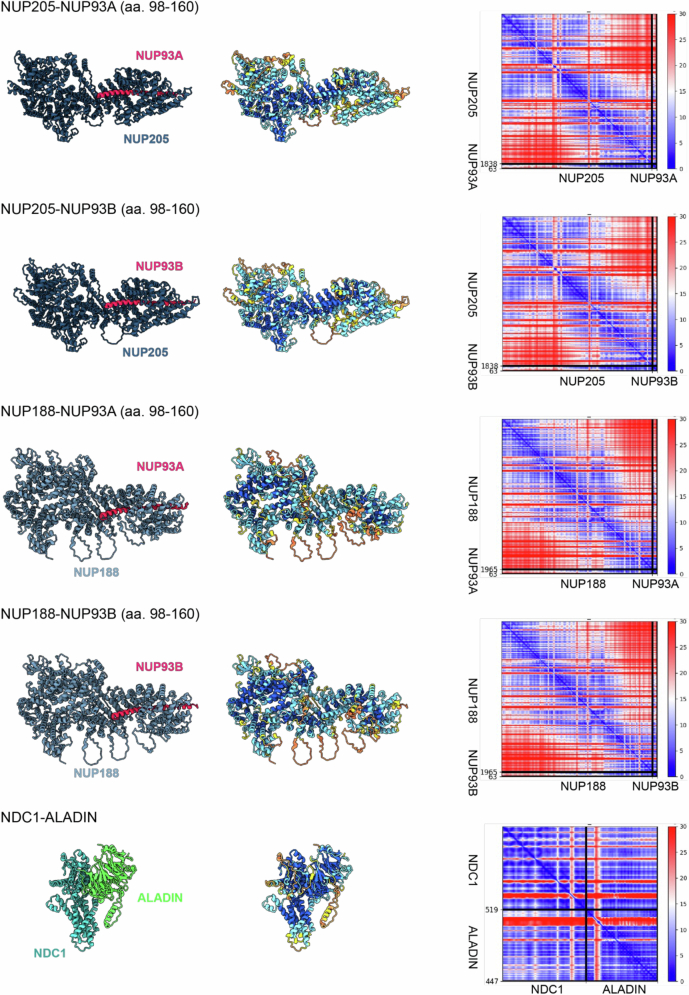

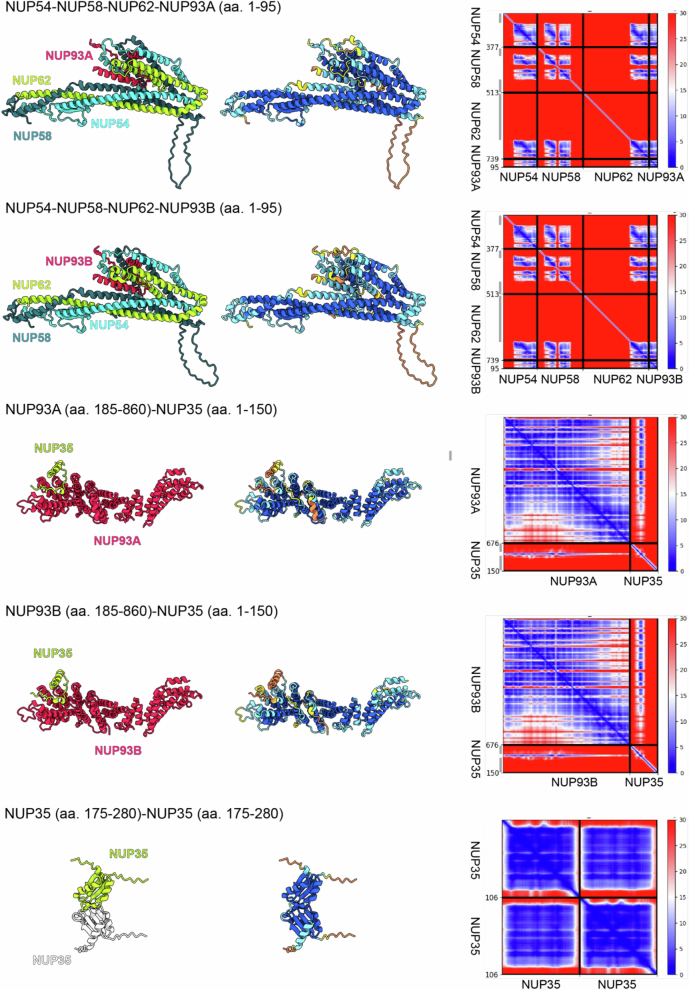

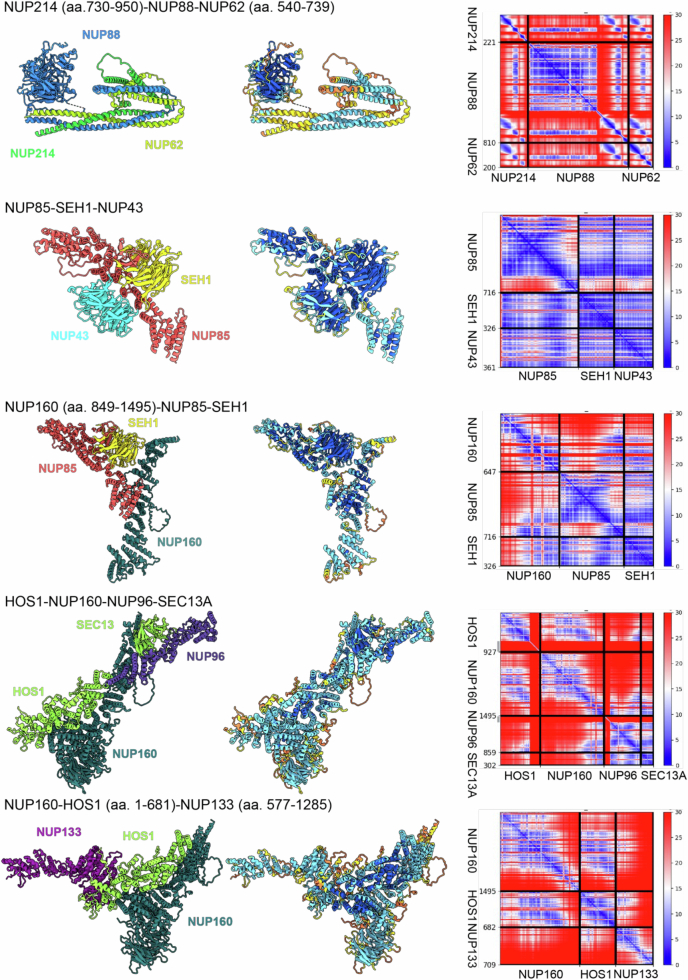

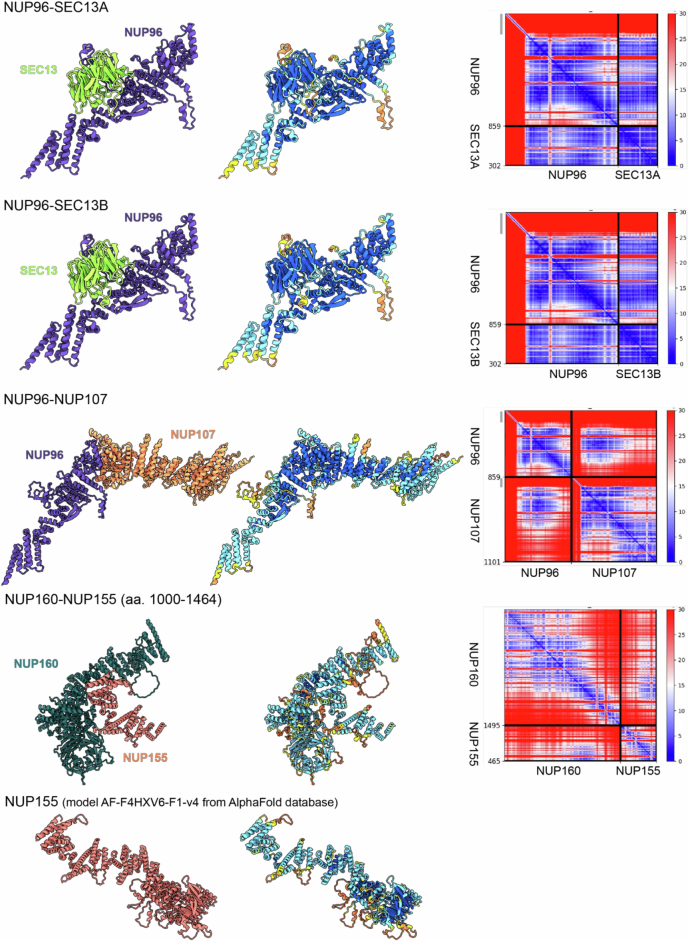

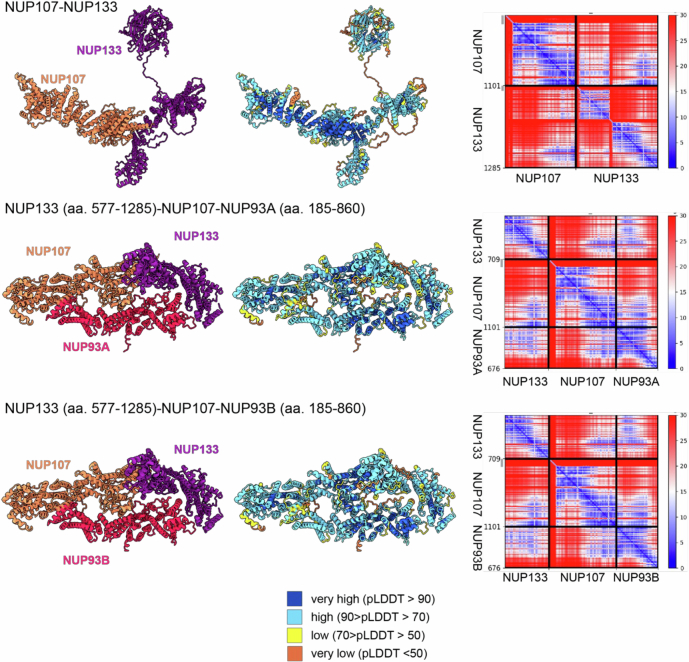
Structural models of the *A. thaliana* NUP subcomplexes modeled using AlphaFold2. Each row shows, from left to right: a model colored by the NUP color code; a model colored by the local confidence estimated with predicted local distance difference test (pLDDT), as returned by AlphaFold. pLDDT > 90 (dark blue) indicates high estimated accuracy of backbone and side chain rotamers, whereas pLDDT > 70 (yellow) indicates confident backbone prediction^54^. The confidence of interdomain and inter-chain orientations estimated with the expected distance error between all pairs of residues in the complex, as returned by AlphaPulldown. The color at each (x, y) position of the matrix corresponds to the expected distance error in residue x’s position when the prediction and true (unknown) structure are aligned on residue y. Blue indicates low error. The heat maps show the sequence covered in the initial prediction and indicated in parenthesis. Long disordered regions, indicated with grey bars next to the matrix are not shown in the figures of model structures. aa. – amino acid residues.

Next, we assessed the AtNPC structural model in further detail and compared it with the available structural model of the human NPC^14^. Unfortunately, we could not include the CrNPC in these further comparisons as no detailed structural model is available. Therefore, we directly compared the AtNPC with the HsNPC structural model and focused on the Y-complexes. The head-to-tail contact in the AtNPC CR Y-complex and NR inner Y-complex is formed between the NUP133 α-solenoid domain and HOS1, while the head-to-tail contact in the human NPC is formed between the NUP133 β-propeller domain and NUP160 (Fig. 4 and Extended Data Fig. 2).

**Fig. 5:**
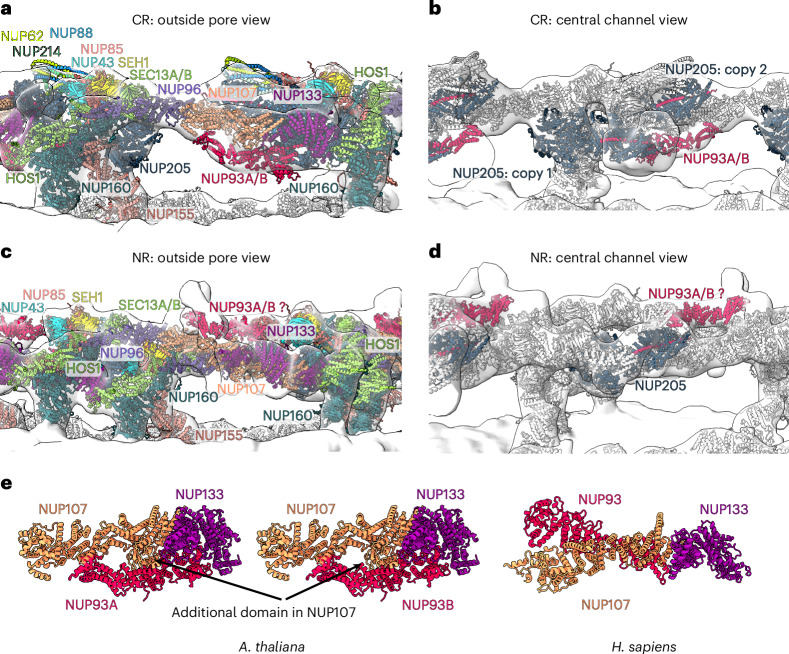
AtNPC architecture. **a**, The CR with NUPs coloured as in Fig. 2. **b**, The CR with two copies of NUP205 and one copy of NUP93A/B shown in the same colour scheme as Fig. 2. **c**, The NR with NUPs coloured as in Fig. 2. **d**, The NR highlighting the NUP205–NUP93A/B complex and the potential position of NUP93A/B coloured as in Fig. 2. **e**, AlphaFold models of the *A.* *thaliana* NUP133–NUP107–NUP93A/B and the *H.* *sapiens* NUP133–NUP107–NUP93 complexes (see also Extended Data Fig. 2).

Contrary to ELYS in the human NPC, one copy of HOS1 appears present in the CR of the AtNPC while two HOS1 copies are present in the NR (Fig. 5a,b). Moreover, two copies of a large NUP, probably representing NUP205, are in the CR of the AtNPC, while only one copy is in the NR (Fig. 5c,d).

The C-terminal domain of NUP93A/B is positioned differently in the AtNPC compared to the C-terminal domain of NUP93 in the HsNPC. There is no density in the AtNPC at the positions corresponding to NUP93 in the HsNPC. The AlphaFold2 model of the interaction between NUP93/NUP93A/B, NUP133 and NUP107 is different in human and *A.* *thaliana*. NUP107 in *A.* *thaliana* has an additional domain (not present in human NUP107) that interacts with NUP93A/B (Fig. 5e). The C-terminal domain of NUP93A/B seems to interact with NUP107 and NUP133 in the CR in the position modelled by AlphaFold2, as it is consistent with the density next to NUP107/NUP133 that would correspond to NUP93A/B. However, NUP93A/B is not present in the corresponding positions in the NR. There is another region in the NR that could possibly be occupied by NUP93A/B (Fig. 5c,d); however, this position of NUP93A/B is speculative since the corresponding interaction was not detected by AlphaFold.

## Discussion

The direct passage of materials through NPCs enables selectivity and the control of molecular trafficking in and out of the nucleus. The high degree of similarity among NPCs across organisms suggests that they evolved from a common ancestor, later developing species-specific features. Despite the crucial importance of plants, technical limitations have hindered a detailed investigation of their NPC structure until recently. Here, we present a detailed three-dimensional structure of the NPC from a higher plant, providing insights into the structural evolution between plant and human NPCs.

Our mass spectrometry results enabled us to compare the NUPs, which are present in AtNPCs, with published data from HsNPC. We identified 31 of the described *A.* *thaliana* NUPs^22,37–39^ in the root protoplasts (Table 1), suggesting that our nuclei purification protocol and mass spectrometry protocol were similarly efficient compared to previously published protocols aiming for NUP identification^39,43^. The techniques used to isolate the nuclear fraction, such as centrifugation, mechanical disruption and exposure to various buffers and reagents, may disrupt the structural integrity of the NPC, leading to the detachment of individual NUPs. While further NUPs have been detected by mass spectrometry in previous studies^37–39,63–67^, their absence could also be explained by our exclusive use of root protoplast cells, whereas other studies utilized whole *A.* *thaliana* plantlets and therefore a mix of different tissue and cell types. Specifically, for NUP136, it is known to be tethered to the plant nucleoskeleton and may have remained attached to proteins performing lamin-like functions^66^. While CG1 has been detected in other published studies^43^, this is probably due to the use of whole *A.* *thaliana* plantlet extracts for NUP detection, which enables the identification of proteins present across multiple tissue types or cell types. For Nup98b it was previously reported that isoforms a and b function redundantly and have a similar temporal expression pattern^65,68^; thus, if we detected unique peptides corresponding to NUP98a, we should have detected unique peptides corresponding to NUP98b. However, this apparent lack of detection of NUP98b is consistent with other proteomic analysis^39^.

With our data and the information about the Y-complex in humans, we were able to determine which elements of the Y-complex are present in both AtNPCs and HsNPCs and which are unique. We show that HsNPC and AtNPC share most NUPs of the Y-complex that are also conserved in other species such as *Saccharomyces cerevisiae*^69^. This highlights their important structural role in NPCs across different species. On the other hand, NUPs ELYS and NUP37 may have evolved to have specific functions in certain species. For example, it was previously reported that HOS1 (detected in several studies^37,70–74^) contains a specific region with homology to ELYS. On the basis of this observation, it was proposed that HOS1 is a functional homologue of ELYS^37^. In *A.* *thaliana*, HOS1 has been shown to indeed interact with NUP160 as would be expected for ELYS. Its interaction with NUP160 and NUP96 in the Y-complex was furthermore proposed to play an important role in flowering regulation in *A.* *thaliana*^59,75^. Consistently, the mislocalization of HOS1 away from the NPC leads to pre-activation of Flowering Locus T and early flowering^75^. In our structural model, we observe HOS1 as a key factor for the head-to-tail interaction of the Y-complex in the NPC, but how this translates to its stability and function remains unclear. In addition, while we observe a clear density for HOS1 in our cryo-ET map from root protoplasts, we cannot exclude that HOS1 may delocalize from NPCs in other cell types or developmental stages, such as proposed for early flowering phenotypes^75^.

Although significant progress has been made in understanding the organization and composition of NPCs in different organisms^10,11,13–15,22,61,76^, research on plants lags behind when compared with other eukaryotic species. Our study closes part of this gap by obtaining a structure of the NPC of a higher plant. Thus, we carried out a comprehensive comparison with NPCs from human, but also compared the overall structure with the more closely related single-cell algae *C.* *reinhardtii*. This analysis enabled us to identify significant distinctions in the structure and dimensions of this large protein complex. By combining subcomplex segmentation with integrative structural modelling using predicted NUP structures and fitting into the in situ cryo-ET map, we provide a comprehensive understanding of its scaffold architecture. This allowed a comparison between AtNPC, CrNPC and HsNPC. Our findings highlight that the AtNPC has structural similarity to the CrNPC and that it is rather conserved with regard to the Y-complex arrangement and their asymmetric distribution across CR and NR. However, NUP155 connector elements between the outer ring and the inner ring are prominent in the AtNPC structure and reminiscent of the connectors in HsNPC, but are absent in the CrNPC. The resemblance between the plant and human connector elements provides an interesting paradox, potentially suggesting that the last common eukaryotic ancestor^77^ would have had prominent connectors and that the shorter connectors, observed in *Chlamydomonas*, could have been acquired through divergent evolution.

The AtNPCs in our protoplast cryo-ET dataset were present with a smaller membrane-to-membrane diameter as compared with the previous HsNPC structure on isolated nuclear envelopes^14^, which were known to be present in a constricted ground state. In cells, several cryo-ET studies of different organisms have shown that NPCs reside in a more dilated state^16,78,79^. Variations in NPC diameter in other organisms have previously been linked to membrane tension, pointing to energy depletion and hyperosmotic response as possible cues for NPC constriction^16,61^. Measurements, obtained using scanning electron microscopy on chemically-fixed and fractured tobacco BY-2 nuclei reported by Fiserova et al., suggested that NPC diameters could fall into different categories, with various internal and external diameters depending on the development state of the cells^80^. It should be noted that with our experimental setup, we only investigate a single timepoint in *A.* *thaliana* development, but we observe that the NPC diameter varies for individual NPCs across our dataset (Extended Data Fig. 1).

### Limitations of the study

While the smaller NPC diameter that we observed could be a characteristic trait of higher plant cells in general, possible adaptations to the AtNPC structure may also be influenced by various exogenous factors: alterations to nuclear envelope membrane tension by taking the root cells out of the tissue context and osmotic effects or perturbations of nuclear transport during the isolation could all affect the NPC diameter and may thus potentially also influence the height of the complex. Since the protoplasts we analysed are isolated cells, we cannot exclude that isolation itself affects NPC diameter and height and that NPCs in cells within the tissue context and other cell types of *A.* *thaliana* seedlings may be affected differently. To perform cryo-ET structural analyses directly in the tissue context is still challenging; however, it might become feasible with advances in cryo-ET sample preparation of plant tissue^81^. This could help to determine the extent to which the AtNPC adapts in the tissue context and during development in future studies.

Our approach of structural modelling into cryo-ET NPC density maps is powerful to assess the overall structure and to fit individual structure predictions and interaction partners of conserved protein subcomplexes. However, the limited resolution of our in situ cryo-ET map also limits the interpretation of our structural model of the AtNPC. For example, we cannot make claims about direct protein–protein interfaces between individual NUPs, which is a constraint that is intrinsic to all current in situ NPC studies^14,16,61,82^. In particular, flexible regions, such as the basket attachment and cytoplasmic filaments, are difficult to assess by cryo-ET at higher resolution and with confidence. Owing to the low numbers of NPCs in the *A.* *thaliana* dataset, we thus refrain from interpretation of these regions.

Despite these limitations, our approach reflects a powerful demonstration of in situ structural biology directly in higher plant cells, leading to valuable new insights about the AtNPC scaffold and its structural conservation compared with unicellular algae and human cells. Our study paves the way for future structural biology studies from plant protoplasts of different tissues to investigate other higher plant organelles and macromolecular complexes.

## Methods

### Plant material and growth conditions

Transgenic *A.* *thaliana* ecotype Columbia (Col-0) expressing green fluorescent protein (GFP) tagged to an RNA export factor (RAE1)^37^ were used in this study. Seeds were surface sterilized with 70% ethanol for 2 min, followed by 7% bleach and 0.1% Triton X-100 for 5 min. Then, seeds were washed four to six times with sterile water and stratified in the dark for 48 h at 4 °C before germination. Seeds were sown at a density of approximately 50 seeds per Petri box over plant growth media consisting of ½ Murashige and Skoog Basal Medium (Sigma-Aldrich) supplemented with 1% agar (Bio Basic) and adjusted to pH 5.7 with KOH (Millipore Sigma). Square Petri dishes were positioned vertically under a long-day photoperiod (16 h of light, 8 h of dark) at 23.5 ± 0.5 °C, with an average light intensity of 120 mmol m^−^^2^ s^−1^ at the level of the rosette.

### Protoplast isolation

Protoplasts from roots were isolated according to ref. ^83^ with minor modifications. Briefly, the enzyme solution containing 0.4 M mannitol (Acros), 20 mM MES pH 5.7 (FisherBiotech), 20 mM KCl (Fisher Scientific), 1.5% Cellulase from *Trichoderma* sp. (Sigma-Aldrich) and 0.3% pectinase from *Aspergillus niger* (Sigma-Aldrich) was warmed at 55 °C for 10 min, then cooled to room temperature. Then, 0.1% BSA, 10 mM of CaCl_2_ (Bio Basic) and 5 mM of β-mercaptoethanol (Fisher Scientific) were added and the resulting solution was filtered through a 0.2 μm syringe filter (Fisher Scientific) into a Petri dish. Root tissue from 14-d-old plants was harvested with a scalpel and deposited and finely chopped in the Petri dish containing the enzyme solution. The enzymatic digestion was performed under agitation at 75 rpm for 1.5 h at room temperature. After cell wall digestion, the solution was filtered through a 30 µm nylon mesh into a Falcon tube. One volume of W5 solution (154 mM NaCl (Fisher Scientific), 125 mM CaCl_2_ (Bio Basic), 5 mM KCl (Fisher Scientific) and 2 mM MES pH 5.7 (FisherBiotech)) was added, and protoplasts were spun for 10 min at 500*g*. Protoplasts were then resuspended in cold W5 solution and quantified under a light microscope and a Neubauer chamber to confirm their integrity. Then, protoplasts were also observed using a wide-field fluorescence microscope and 50 µl of Concanavalin A (concentration 1 mg ml^−1^) to a clean glass-bottom dish to help immobilize the protoplasts while imaging them to assess GFP fluorescence and protoplast integrity.

### Nuclei isolation

Nuclei from root protoplasts were obtained according to the method described in ref. ^36^ with some modifications. The whole procedure was carried out at 4 °C. Briefly, cold Nuclear Isolation Buffer (NIB; 10 mM MES KOH pH 5.5, 0.2 M sucrose, 2.5 mM EDTA, 2.5 mM dithiothreitol, 0.1 mM spermine, 10 mM NaCl, 10 mM KCl and 0.15% Triton X-100) was added into a 50 ml round-bottomed centrifuge tube containing no more than 15 × 10^6^ protoplasts per 15 ml. Deplasmolysis was enabled by keeping the NIB containing the protoplasts at 4 °C for 7 min. The entire solution was then passed four times through a 26ga (brown) syringe needle. The solution containing the broken protoplasts was then passed through a 30 µm mesh. The filtered solution was centrifuged at 1,000*g* for 8 min at 4 °C and the white pellet containing the nuclei was collected. For later usage, the nuclei were kept in aliquots in 1.5 ml Eppendorfs with NIB + 20% glycerol at −80 °C. In total, three biological replicates were obtained for further analysis by mass spectrometry.

### Acetone precipitation

Protein precipitation for mass spectrometry was carried out based on a protocol provided by The Proteomics Platform of the Quebec Genomics Center at the CHU de Québec Research Center. Briefly, the samples containing the nuclear extracts were transferred to 2 ml Eppendorf tubes. To each of the samples, four volumes of acetone were added at −20 °C and then vortexed. Samples with acetone were incubated at −20 °C overnight. Then, the samples were centrifuged at 16,000*g* for 15 min at 4 °C and the supernatant was discarded. The tubes containing the pellet with the nuclear extracts were dried under a hood for 5 min to let the acetone evaporate and the samples were resuspended in a protein extraction buffer containing 50 mM ammonium bicarbonate and 1% sodium deoxycholate.

### Mass spectrometry

Nuclear extract samples were sent for analysis to the Proteomics platform of the CHU de Québec Research Center to detect the presence of NUPs. Using dithiothreitol (0.2 mM at 37 °C for 30 min), iodoacetamide (0.8 mM at 37 °C for 30 min) and trypsin (0.2 µg at a ratio of 1:50 protease:protein, 37 °C overnight incubation), samples were reduced, alkylated and digested, respectively. Tryptic peptides were desalted, vacuum dried and resuspended in a 0.1% formic acid solution. Peptide quantities were calculated using a Nanodrop (205 nm absorbance). A Dionex UltiMate 3000 nanoRSLC chromatography system (Thermo Fisher Scientific) coupled to an Orbitrap Fusion mass spectrometer (Thermo Fisher Scientific) was used for analysing samples (1.0 µg) by nano liquid chromatography–tandem mass spectrometry. Peptides were separated on a Pepmap Acclaim (Thermo Fisher) 50 cm × 75 µm internal diameter separation column, using 300 nl min^−1^ for a 90 min linear gradient from 5% to 40% solvent B (A, 0.1% formic acid; B, 80% acetonitrile, 0.1% formic acid). Thermo XCalibur software version 4.1.50 was used to collect mass spectra using the data-dependent acquisition method. Precursor ions were analysed in an Orbitrap at a resolution of 120 000 *m*/*z* and the most intense ions were selected for higher-energy collisional dissociation fragmentation by a quadrupole analyser using 1.6 *m*/*z* isolation windows followed by fragment mass scans in an Ion Trap, with a method set to a maximum cycle time of 3 s.

### Database searching

Thermo raw spectrum files were converted to MGF peak list file format by Proteome Discoverer 2.3 (Thermo Fisher Scientific). Filtered tandem mass spectrometry data were then analysed using Mascot (Matrix Science, version 2.5.1). Mascot was set up to search against a UniProt *A.* *thaliana* Reference proteome database (UP000006548, version at 24 August 2020). Mascot search parameters included a 0.60 Da fragment ion mass tolerance and a 10.0 ppm parent ion tolerance. Cysteine carbamidomethylation was set as a fixed modification while deamidation of asparagine and glutamine as well as the oxidation of methionine were set as variable modifications.

### Protein identification and data analysis

Scaffold software (version 5.2.2, Proteomes Software Inc.) was used to validate tandem mass spectrometry-based peptide and protein identification. Peptide identification was accepted if the Scaffold local FDR method could establish it with a higher than 91.0% probability to achieve an FDR less than 1.0%. Proteins that included at least two known peptides and could be identified with a probability of more than 99.0% to obtain an FDR less than 1.0% were accepted. The Protein Prophet program was used to assign a probability for each protein^84^. To adhere to the parsimony criteria, proteins with identical peptide compositions that could not be distinguished based only on tandem mass spectrometry analysis were categorized. Data were considered using exponentially modified protein abundance index (emPAI) values^85^. The mass spectrometry data generated in this study have been deposited in the Proteomexchange database under accession code PXD061805.

### Y-complex NUPs comparison

All PDB AlphaFold files of NUPs from *A.* *thaliana* and *H.* *sapiens* were visualized using the PyMOL Molecular Graphics System, version 4.6 (Schrödinger LLC).

### TM score alignment values

TM alignment for protein structure comparison was performed using the TM-align tool from the Zhang Lab group^56^. Alphafold IDs and PDB files for the NUPs compared were obtained from the AlphaFold Protein Structure Database (https://alphafold.ebi.ac.uk/).

### Cryo-EM sample preparation and cryo-CLEM

Cryo-EM grid preparation of root protoplasts for data acquisition were prepared as described earlier^25^. Briefly, using a plunge freezer (Leica GP2), set to 70% humidity, single back-side blotting, 6 s blot time and no delay before blotting, EM grids (Au grids 200 mesh, SiO_2_ foil, R2/1 from Quantifoil) were glow discharged two times with a Pelco easiGlow glow discharger for 90 s at 15 mA each. Root protoplasts were adjusted to a concentration of 500–650 protoplasts per µl in fresh W5 solution. A 3 µl droplet containing 1,500–1,950 protoplasts was back-side blotted with Whatman filter paper grade 1 for 6 s and vitrified onto each EM grid using a Leica Plunge Freezer EM GP2 by plunge freezing into liquid ethane. The frozen grids were clipped and imaged on an EM cryo-CLEM system (Leica Microsystems). Imaging was performed using a HC PL APO 50×/0.90 DRY objective, 488 nm laser excitation and detecting simultaneously at 498–542 nm.

### Cryo-FIB milling

Lamellae from plunge-frozen grids were prepared with an Aquilos FIB–SEM microscope (Thermo Scientific) similarly to previous protocols^13,86^. Briefly, samples were coated with a layer of organometallic platinum for 10 s with the gas injection system. Then they were additionally sputter coated with platinum for 10 s at 1 kV and 10 mA. Lamellae milling was performed in a step-wise fashion by decreasing the FIB current from 1 nA, 500 pA and 300 pA to 100 pA. The final polishing of the lamellae was carried out with 30–50 pA to a final thickness of ~180–200 nm. Finally, an additional sputter layer of platinum at 1 kV and 10 mA was added for 1–2 s before unloading the sample.

### Cryo-ET acquisition

A total of 111 tilt series were acquired in three independent microscope sessions on a Titan Krios G2 Cryo-Transmission Electron Microscope (Thermo Scientific), operating at 300 kV and equipped with a BioQuantum-K3 (Gatan) imaging filter. Before tilt-series acquisition, the autogrids were carefully loaded with the lamella orientation perpendicular to the tilt axis of the microscope. Using SerialEM (version 3.8.1) in low-dose mode, tilt series were recorded as 6 K × 4 K movies with 10 frames each, and motion-corrected in SerialEM on-the-fly. Projection images had a magnification of 42,000×, corresponding to a unbinned pixel size of 2.176 Å. Acquisition of tilt series began either at 0° or with a lamella pre-tilt of −8°. A dose-symmetric acquisition strategy with 2° increments was used, yielding approximately 60 projections per tilt series with a constant exposure time, totalling ~130 e^−^ Å^−^^2^. The targeted defocus was adjusted from −2.5 to −5 μm, while the energy slit width was fixed at 20 eV. The detector dose rate was intended to be ~14.5 e^−^ px^−1^ s^−1^ at lamella pre-tilt.

### Tilt-series processing

All images were preprocessed by dose filtering using MATLAB as described previously^87^. From the dose-filtered tilt series, poor-quality tilt images were removed after visual inspection. Using the etomo program in IMOD^88^, dose-filtered tilt series were then aligned with the patch tracking^88,89^ and reconstructed as back-projected tomograms using SIRT-like filtering at a binned pixel size of 8.704 Å. Based on the thickness of the reconstructed tomograms and quality of patch tracking, features contained in the volume 37 were chosen for further STA after visual assessment. For NPC STA, 3D CTF-corrected back-projected tomograms were generated using NovaCTF^90^.

### STA

A total of 79 NPCs were manually selected from four-times binned tomograms. NPC coordinates and initial orientations were manually selected in tomograms that were filtered in a SIRT-like fashion^16,91^. Initial alignment of NPCs was carried out on whole NPCs^16,53^. All alignment steps were performed using novaSTA^90^. The coordinates of NPC spokes were determined based on eightfold symmetry after establishing an initial four-times binned whole NPC map, as previously described^16^. A mask covering each asymmetric unit (CR, IR and NR) was used for further alignment. Each subtomogram and its assigned orientation were manually examined following initial subunit alignment, and any misaligned or out of lamella particles were eliminated^91,92^. Four-times binned subtomograms and tight masks were used for focused alignment on the CR, IR or NR. The final individual ring maps were first fitted to the STA map of the whole asymmetric subunit and a whole NPC composite map was created based on the eightfold symmetry of the NPC.

### NPC diameter measurements

Based on the final coordinates and orientations acquired during STA, NPC diameters were calculated using a MATLAB script used in previous studies^16^. Only NPCs with a subunit occupancy of five or more were used when calculating the diameter of an NPC. Distances linking the opposing components were calculated for each individual NPC. The intersection point of all the vectors related to a certain NPC was used to establish the centre of each NPC based on those distances. The typical NPC radius for a pore was determined to be the average distance between the newly found centre and each individual subunit. This method allowed the evaluation of the average radius for a particular characteristic of interest inside each NPC.

### Structural modelling of *A. thaliana* NUPs and NPC subcomplexes

The structures of individual NUPs and NPC subcomplexes were modelled using AlphaFold2 (refs. ^54,93^) available through AlphaPulldown^94^. The max_recycles parameter was set to 48. The following models were generated: NUP205–NUP93A (amino acid (aa) 98–160), NUP205–NUP93B (aa 98–160), NUP188–NUP93A (aa 98–160), NUP188–NUP93B (aa 98–160), NDC1–ALADIN, NUP54–NUP58–NUP62–NUP93A (aa 1–95), NUP54–NUP58–NUP62–NUP93B (aa 1–95), NUP93A (aa 185–860)–NUP35 (aa 1–150), NUP93B (aa 185–860)–NUP35 (aa 1–150), NUP35 (aa 175–280)–NUP35 (aa 175–280), NUP214 (aa 730–950)–NUP88–NUP62 (aa 540–739), NUP85–SEH1–NUP43, NUP160 (aa 849–1495)–NUP85–SEH1, HOS1–NUP160–NUP96–SEC13A, NUP160–HOS1 (aa 1–681)–NUP133 (aa 577–1,285), NUP96–SEC13A, NUP96–SEC13B, NUP96–NUP107, NUP160–NUP155 (aa 1,000–1,464), NUP107–NUP133, NUP133 (aa 577–1,285)–NUP107–NUP93A (aa 185–860) and NUP133 (aa 577–1,285)–NUP107–NUP93B (aa 185–860) (Extended Data Fig. 2).

### Fitting of the AlphaFold models into cryo-ET maps

To generate the model of the asymmetric unit of the AtNPC, we used the model of the human NPC (PDB 7R5J)^14^ as a template. We fitted the IR and the fragments of CR and NR of the human NPC into the map of the AtNPC. Then, we superposed AlphaFold models of the AtNPC subcomplexes to the human model and optimized the fits of the AtNPC subcomplexes into the map of the AtNPC using ChimeraX^95^.

### Modelling of the AtNPC scaffold

To assemble the model of the entire NPC scaffold we used the integrative modelling software Assembline^96^, which is based on Integrative Modeling Platform version 2.15^97^ and Python Modeling Interface^98^.

In addition to using models of subcomplexes as rigid bodies for fitting in the modelling, several intersubunit interfaces were restrained by elastic distance network derived from AlphaFold models, overlapping and bridging the already fitted models. During the refinement, the structures were used as rigid bodies and simultaneously represented at two resolutions: Cα-only representation and a coarse-grained representation, in which ten-residue fragments were represented as a single bead. The Cα-only representation was used for all restraints except for the EM fit restraint.

The NPC structure was optimized using the refinement protocol of Assembline to optimize the fit to the map, minimize steric clashes and ensure connectivity of the protein linkers. The scoring function for the refinement comprised the EM fit restraint, clash score (SoftSpherePairScore of Integrative Modeling Platform), connectivity distance between domains neighbouring in sequence and elastic network restraints derived from the subcomplexes modelled with AlphaFold.

### Reporting summary

Further information on research design is available in the Nature Portfolio Reporting Summary linked to this article.

## Supplementary information

Reporting Summary

## Data Availability

The mass spectrometry data generated in this study have been deposited in the Proteomexchange database under accession code PXD061805. The AtNPCs maps reported in this study are available through the EM Data Bank with accession codes EMD-54653, EMD-54654, EMD-54655 and EMD-54656. The composite AtNPC is available at EMD-54657. The modelled AtNPC structure of this study is available at PDB 9SOB. Cryo-ET maps of CrNPC EMD-(4355) and the HsNPC EMD-(14321) reported in previous studies are available through EMDB. The HsNPC model of a previous study is available at PDB 7R5J.
