# Supplementary material for: In situ architecture of the nuclear pore complex of the higher plant Arabidopsis thaliana
Source: Nat Plants. 2025 Oct 31;11(11):2368–80. doi: 10.1038/s41477-025-02138-y (PMC12626886; doi:10.1038/s41477-025-02138-y)
Supplement: Supplementary file 1 — Reporting Summary [file 41477_2025_2138_MOESM1_ESM.pdf]

nature portfolio

Corresponding author(s): Hugo Germain

Last updated by author(s): Mar 25, 2025

Reporting Summary

Nature Portfolio wishes to improve the reproducibility of the work that we publish. This form provides structure for consistency and transparency in reporting. For further information on Nature Portfolio policies, see our Editorial Policies and the Editorial Policy Checklist.

Statistics

For all statistical analyses, confirm that the following items are present in the figure legend, table legend, main text, or Methods section.

n/a	Confirmed	
☐	☒	The exact sample size (*n*) for each experimental group/condition, given as a discrete number and unit of measurement
☐	☒	A statement on whether measurements were taken from distinct samples or whether the same sample was measured repeatedly
☒	☐	The statistical test(s) used AND whether they are one- or two-sided<br>*Only common tests should be described solely by name; describe more complex techniques in the Methods section.*
☒	☐	A description of all covariates tested
☒	☐	A description of any assumptions or corrections, such as tests of normality and adjustment for multiple comparisons
☐	☒	A full description of the statistical parameters including central tendency (e.g. means) or other basic estimates (e.g. regression coefficient) AND variation (e.g. standard deviation) or associated estimates of uncertainty (e.g. confidence intervals)
☒	☐	For null hypothesis testing, the test statistic (e.g. *F*, *t*, *r*) with confidence intervals, effect sizes, degrees of freedom and *P* value noted<br>*Give P values as exact values whenever suitable.*
☒	☐	For Bayesian analysis, information on the choice of priors and Markov chain Monte Carlo settings
☒	☐	For hierarchical and complex designs, identification of the appropriate level for tests and full reporting of outcomes
☒	☐	Estimates of effect sizes (e.g. Cohen's *d*, Pearson's *r*), indicating how they were calculated

Our web collection on statistics for biologists contains articles on many of the points above.

Software and code

Policy information about availability of computer code

Data collection	Data collection was performed by using SerialEM software (please see Methods in the manuscript)
Data analysis	Software used is described in Methods section of the manuscript:<br>Scaffold software (version 5.2.2, Proteomes Software Inc., Portland, USA), PyMOL Molecular Graphics System, Version 4.6 Schrödinger, LLC., SerialEM (version 3.8.1), IMOD (versions 4.10.9 and 4.11.5), NovaSTA, MATLAB R2019b, Excel (2021), AlphaFold2, ChimeraX (1.8-1.10), Assembline based on Integrative Modeling Platform (IMP) version 2.15 92, Python 555 Modeling Interface (PMI), AlphaFold

For manuscripts utilizing custom algorithms or software that are central to the research but not yet described in published literature, software must be made available to editors and reviewers. We strongly encourage code deposition in a community repository (e.g. GitHub). See the Nature Portfolio guidelines for submitting code & software for further information.

Data

Policy information about availability of data

All manuscripts must include a data availability statement. This statement should provide the following information, where applicable:

- Accession codes, unique identifiers, or web links for publicly available datasets
- A description of any restrictions on data availability
- For clinical datasets or third party data, please ensure that the statement adheres to our policy

The mass spectrometry data generated in this study have been deposited in the Proteomexchange database under accession code PXD061805 and are publicly

accessible. The cryo-ET AtNPCs maps reported in this study are available through the EM Data Bank with accession codes EMD-54653, EMD-54654, EMD-54655, EMD-54656. The composite AtNPC is available as EMD-54657. The modeled A. thaliana NPC structure of this study is being made available at PDB 9SOB. Cryo-ET maps of C. reinhardtii NPC (EMD-4355) and the H. sapiens NPC (EMD-14321) reported in previous studies are available through EMDB. The H. sapiens NPC model of a previous study is available as PDB 7R5J.

Research involving human participants, their data, or biological material

Policy information about studies with human participants or human data. See also policy information about sex, gender (identity/presentation), and sexual orientation and race, ethnicity and racism.

Reporting on sex and gender	Manuscript does not involve the participation of humans
Reporting on race, ethnicity, or other socially relevant groupings	Manuscript does not involve the participation of humans
Population characteristics	Manuscript does not involve the participation of humans
Recruitment	Manuscript does not involve the participation of humans
Ethics oversight	Manuscript does not involve the participation of humans

Note that full information on the approval of the study protocol must also be provided in the manuscript.

Field-specific reporting

Please select the one below that is the best fit for your research. If you are not sure, read the appropriate sections before making your selection.

☒ Life sciences ☐ Behavioural & social sciences ☐ Ecological, evolutionary & environmental sciences

For a reference copy of the document with all sections, see nature.com/documents/nr-reporting-summary-flat.pdf

Life sciences study design

All studies must disclose on these points even when the disclosure is negative.

Sample size	There was not an specific sample size to achieve when designing the experiments. Instead, the quantities of samples and data acquired where the maximum available within the equipment used.
Data exclusions	A portion of the cryoET data that was acquired needed to be excluded due to low quality that could lead to bad alignment of the same data, as well as bad reconstruction of it.
Replication	Three independent cryo-ET data collections were carried out independently of each other over multiple cells.<br>As well, grid samples were prepared from separately isolated root protoplasts. These results confirmed the reproducibility of the workflow. To maximize the signal to noise and resolution of the cryo-ET maps, all three data collections were combined.
Randomization	However, for the resolution estimation using fourier shell correlation, subtomogram averaging was performed for two separate halfsets. For other aspects of the study, we not perform randomization.
Blinding	The investigators were not blinded during data collection and analysis because data was not grouped for analysis. For FSC resolution estimation, subtomograms lists were split into even and odd particle numbers.

Reporting for specific materials, systems and methods

We require information from authors about some types of materials, experimental systems and methods used in many studies. Here, indicate whether each material, system or method listed is relevant to your study. If you are not sure if a list item applies to your research, read the appropriate section before selecting a response.

Materials & experimental systems

n/a	Involved in the study
☒	☐ Antibodies
☒	☐ Eukaryotic cell lines
☒	☐ Palaeontology and archaeology
☒	☐ Animals and other organisms
☒	☐ Clinical data
☒	☐ Dual use research of concern
☐	☒ Plants

Methods

n/a	Involved in the study
☒	☐ ChIP-seq
☒	☐ Flow cytometry
☒	☐ MRI-based neuroimaging

Dual use research of concern

Policy information about dual use research of concern

Hazards

Could the accidental, deliberate or reckless misuse of agents or technologies generated in the work, or the application of information presented in the manuscript, pose a threat to:

No	Yes
☒	☐ Public health
☒	☐ National security
☒	☐ Crops and/or livestock
☒	☐ Ecosystems
☒	☐ Any other significant area

Experiments of concern

Does the work involve any of these experiments of concern:

No	Yes
☒	☐ Demonstrate how to render a vaccine ineffective
☒	☐ Confer resistance to therapeutically useful antibiotics or antiviral agents
☒	☐ Enhance the virulence of a pathogen or render a nonpathogen virulent
☒	☐ Increase transmissibility of a pathogen
☒	☐ Alter the host range of a pathogen
☒	☐ Enable evasion of diagnostic/detection modalities
☒	☐ Enable the weaponization of a biological agent or toxin
☒	☐ Any other potentially harmful combination of experiments and agents

Plants

Seed stocks	Arabidopsis thaliana RAE1-GFP seeds were obtained from Kentaro Tamura
Novel plant genotypes	No novel plant genotypes were produced
Authentication	*Describe any authentication procedures for each seed stock used or novel genotype generated. Describe any experiments used to assess the effect of a mutation and, where applicable, how potential secondary effects (e.g. second site T-DNA insertions, mosiacism, off-target gene editing) were examined.*
